# Psychometric evaluation of the DSM-5-TR-Level 1 Cross-Cutting Symptom Measure: transdiagnostic factor analysis in a real-world psychiatric outpatient sample

**DOI:** 10.3389/fpsyt.2026.1680352

**Published:** 2026-04-10

**Authors:** Carlene MacMillan, Nitin Gogtay, Debbie Gibson, Jimmy J. Qian, William M. Sauve, Emily Shih

**Affiliations:** 1 Osmind Inc., San Francisco, CA, United States; 2 Radial Health, New York, NY, United States; 3 American Psychiatric Association, Washington, DC, United States

**Keywords:** confirmatory factor analysis, DSM-5-TR-Level 1 Cross-Cutting Symptom Measure, exploratory factor analysis, factor analysis, general psychopathology, mental disorders, outpatients, real-world evidence

## Abstract

**Background:**

Dimensional approaches to psychiatric assessment are increasingly used to complement categorical diagnoses. The DSM–TR Level 1 Cross-Cutting Symptom Measure (DSM-XC) assesses symptoms across psychopathology domains, but its latent structure remains unexplored in large, real-world psychiatric outpatient populations, limiting guidance on how clinicians interpret and apply results.

**Methods:**

We analyzed real-world data from patients (*N* = 3,101) using the Osmind platform who completed the DSM-XC as part of measurement-based care (the systematic collection of patient-reported outcomes to guide clinical decision-making). Exploratory factor analysis was conducted in half of the sample, and confirmatory factor analysis tested two models in the remaining half: a 6-factor general model, a 5-factor bifactor model with a general psychopathology factor. A third confirmatory model based on Caspi et al’s framework was also evaluated using the full sample.

**Results:**

The 6-factor general model identified domains of ‘Mood’, ‘Anxiety’, ‘Depression’, ‘Psychosis’, ‘Substance Use’, and ‘Distress and Disconnection’. The 5-factor bifactor model demonstrated a strong general psychopathology factor. The Caspi-inspired model showed acceptable fit but weaker support for the specific dimensions. These results underscore the DSM-XC’s capacity to capture both shared and domain-specific dimensions of psychopathology, highlighting its relevance for dimensional assessment.

**Conclusion:**

By clarifying its latent structure, this study identified clinically meaningful symptom dimensions which helps facilitate interpretation and application of the DSM-XC in the real-world. As dimensional and measurement-based approaches continue to expand in psychiatry, clearer understanding of tools such as the DSM-XC may support their integration into clinical practice and future research.

## 1 Introduction

Mental health disorders are among the leading causes of disability worldwide, placing a substantial burden on individuals, families, and healthcare systems (e.g., 1, 2). Despite their prevalence and impact, diagnostic practices have remained largely unchanged. Traditionally, psychiatric diagnoses have relied on categorical approaches, such as those outlined in the Diagnostic and Statistical Manual of Mental Disorders (DSM). While these diagnostic categories have provided a foundation for clinical practice and research, they often fail to capture the dimensional and transdiagnostic nature of psychopathology (e.g., 3, 4). Growing evidence suggests that many mental health symptoms cut across diagnostic categories, reflecting shared vulnerabilities and overlapping presentations (e.g., 5–7). In response, dimensional models have emerged as a promising alternative, emphasizing symptom severity and shared factors across diagnostic categories rather than discrete boundaries between disorders (e.g., 8, 9). This shift is particularly relevant given the high comorbidity rates among psychiatric disorders (e.g., 10), which categorical approaches struggle to address.

Despite these aspects of psychiatric illness, widely used mental health measures, such as the Patient Health Questionnaire 9 for depression (11) and the Generalized Anxiety Disorder 7 for anxiety (12), remain narrowly focused on single diagnostic domains. Such instruments, while clinically useful, provide limited insight into the broader symptom landscape experienced by many patients. To address these limitations, the American Psychiatric Association (13) developed the DSM-TR Level 1 Cross-Cutting Symptom Measure (DSM-XC). The DSM-XC is a 23-item patient-reported screener designed to assess symptoms across 13 transdiagnostic domains, including depression, anxiety, mania and psychosis, personality disorders, and substance use (e.g., 14). The DSM-XC is intended as a brief Level-1 screener to be administered across adult patients, with follow-up Level 2 measures used for more targeted assessment when clinically indicated. Its broad scope and dimensional orientation make it a potentially valuable tool for clinical and research settings, particularly in cases where patients present with complex and overlapping symptom profiles.

Despite its potential, limited research has explored the measure’s latent dimensionality, particularly in real-world clinical populations. Characterizing the underlying factor structure is important because it helps determine whether the items reflect coherent and clinically meaningful symptom dimensions, which informs interpretation of screening results and the measure’s utility in guiding targeted Level 2 assessments. Prior studies have relied on translated versions (e.g., 15), focused on non-clinical or specific subpopulations (e.g., 16, 17), or been constrained by small sample sizes (e.g., 18). These methodological limitations often prevent robust factor analytic approaches, leaving critical questions about the measure’s structure and clinical utility unanswered. To date, only one study has evaluated the DSM-XC using a sufficiently large sample for factor analysis: Gibbons and colleagues (19) examined a non-clinical convenience sample (*N* = 3,533) and found that the DSM-XC functions as a multidimensional rather than a unidimensional measure. They identified two well-fitting solutions. The first was a 6-factor model comprising domains labeled ‘Mood’, ‘Worry’, ‘Activation’, ‘Somatic Symptoms’, ‘Thought Disturbance’, and ‘Substance Use’. The second was a 3-factor bifactor model consisting of a general psychopathology factor and two residual factors labeled ‘Internalizing’ and ‘Thought Disorder’. Additionally, a previously proposed two-factor model (20) demonstrated acceptable fit, providing support for earlier work. Collectively, these findings support a multidimensional conceptualization of the DSM-XC. While this study provided important early insights, their sample, recruited online through email, social media, and other electronic methods, was not a clinical population. To be eligible, participants were only required to be 18 years old or older and English-speaking. Additionally, Question 11 (self-harm) was excluded due to the inability to follow up with at-risk individuals. As a result, the generalizability of these findings to psychiatric care settings remain uncertain. Understanding the DSM-XC’s structure in real-world clinical populations is therefore an important next step for determining its utility as a dimensional assessment tool in psychiatric care.

One transdiagnostic framework that has gained traction in the field is Caspi et al’s (5) model of psychopathology, which organizes mental disorders into three broad domains: internalizing (e.g., mood and anxiety-related symptoms), externalizing (e.g., impulsivity, substance use, and behavioral dysregulation); and thought dysfunction (e.g., symptoms of psychosis and cognitive disorganization). These domains are nested within a higher-order general psychopathology factor (“p factor”), reflecting overall liability to mental illness. Caspi’s framework provides a compelling lens through which to evaluate whether both a general psychopathology (“p-factor”) and specific symptom dimensions can be identified in DSM-XC data. Although this framework has been supported in epidemiologic and longitudinal cohorts, it remains unclear whether a brief, clinically administered measure such as the DSM-XC captures comparable dimensions in real-world outpatient populations.

Taken together, these gaps highlight the need to evaluate the latent structure of the DSM-XC in real-world clinical populations and to examine the extent to which it reflects established dimensional and transdiagnostic models of psychopathology. The present study sought to address these gaps. Using factor analytic methods, we pursued two primary aims. First, we conducted exploratory analyses to identify the underlying symptom dimensions represented by the DSM-XC in an outpatient psychiatric sample and to evaluate both general and bifactor solutions, with the latter used to test for the presence of a general psychopathology factor. Second, we tested the clinical utility of a theoretically specified bifactor model aligned with Caspi’s transdiagnostic framework, examining whether internalizing, externalizing, and thought dysfunction domains could be meaningfully recovered within this measure. These findings have important implications for the interpretation and application of the DSM-XC in clinical settings.

## 2 Methods

The study used a cross-sectional observational design to evaluate the latent factor structure of the DSM-XC in a large-real world outpatient psychiatric sample. Using routine clinical data, we applied an exploratory-confirmatory analytic framework in which exploratory factor analysis (EFA) was first used to identify candidate structures and then confirmatory factor analysis (CFA) was used to formally evaluate competing models in an independent subsample. Models included multidimensional and bifactor specifications to evaluate both domain-specific and general psychopathology factors. Analytic decisions were guided by established psychometric principles, model fit indices, and clinical interpretability (e.g., 21, 22).

### 2.1 Patient sample & procedure

The study utilized de-identified, real-world data derived from the Osmind electronic health records (EHR) system. The sample included 3,101 adults who submitted at least one DSM-XC entry on or before January 2025, treated across 169 private psychiatric outpatient practices in 48 states. Most participants were female (63%, *n* = 1,959), with 31% male (*n* = 966) and 6% undisclosed. The racial distribution was predominantly Caucasian (*n* = 2,020; 65%), followed by Asian (*n* = 118; 4%), Mixed race (*n* = 110; 4%), Black (*n* = 109; 4%), and American Indian/Alaska Native (*n* = 18; <1%), with 23% (*n* = 700) choosing not to disclose their race. The mean age was 38.8 years (*SD* = 13.8). Common diagnoses in this sample included major depression (63%), generalized anxiety disorder (57%), and ADHD (37%), with an average of 6 psychiatric comorbidities, reflecting the complexity typical in outpatient psychiatric care.

The DSM-XC was implemented within a Learning Health System (LHS) designed to integrate EHR with real-world evidence generation. As part of routine outpatient care, patients were asked to complete the DSM-XC during standard psychiatric evaluations, based on clinician judgement. Administration of patient-reported measures was scheduled and managed within the EHR, and responses were recorded directly in the system. For the present analysis, only the first DSM-XC assessment per patient was included to avoid bias from repeated measures, as longitudinal analyses were beyond the scope of this study. Clinicians began administering the DSM-XC after it became available in the platform and incorporated it into care at varying points in patients’ treatment courses. As such, the timing of the first observed assessment relative to treatment initiation varied across patients. Patients in this cohort had a mean of 7.42 treatment sessions (*SD* = 11.3; median = 4) prior to their first observed DSM-XC assessment.

Patients consented to research use of de-identified data when registering for the Osmind EHR platform, with the option to opt out. All data were securely stored and de-identified prior to research access. The final dataset included demographics, mental health history, and first-available DSM-XC responses. The study protocol was deemed exempt by the Western Copernicus Group (WCG) Institutional Review Board.

### 2.2 Measures

The DSM-XC is a comprehensive measure designed to assess the severity of various psychiatric symptoms across 13 domains: depression, anger, mania, anxiety, somatic smptoms, suicidal ideation, psychosis, insomnia, memory, repetitive thoughts and behaviors, dissociation, personality functioning, and substance use. The items ask patients to report how much (or how often) they have been bothered by each issue over the past two weeks. Responses are categorized into five levels: not at all (none), rare, less than a day or two (slight), several days (mild), more than half the days (moderate), and nearly every day (severe). The DSM-XC functions as a screening tool; responses rated 2 or higher or affirmative yes/no responses are considered clinically relevant and generally prompt follow-up with the corresponding DSM-5 Level 2 measures. Internal consistency was assessed in the full analytic sample using Cronbach’s alpha for the total scale and multi-item domains. The total scale demonstrated good reliability (*ɑ* = .90), and coefficients for multi-item domains ranged from .36 to .87 (refer to **Supplementary Table 3**).

### 2.3 Statistical analysis

The dataset was first analyzed using descriptive statistics to summarize the demographic and clinical characteristics of the sample. We also detailed the highest and lowest endorsed DSM-XC items to highlight the most and least frequently reported symptoms in the sample.

For Aim 1, the dataset was randomly split into two equal subsamples to facilitate model development and evaluation. EFA was first conducted on one subsample (*n* = 1,550) to uncover the latent structure of the DSM-XC. The analysis used diagonally weighted least squares (DWLS) estimation, which is robust to non-normality (e.g., 23). The ‘geomin’ and ‘bi-geomin’ rotation methods were applied to help identify factors that best represent the underlying symptom dimensions (e.g., 24), and the Satorra-Bentler correction was applied to the test statistic (e.g., 25, 26). General factor solutions ranging from one to eight factors and bifactor models ranging from two to eight factors were evaluated, yielding 15 candidate models. The bifactor models were included to explicitly test the presence of a general psychopathology factor alongside more specific symptom clusters. Item retention was guided by a combination of statistical and substantive criteria (e.g., 27). Items with salient loadings (≥.30; 28) were retained, and loadings slightly below this threshold were considered when conceptually central to a factor. Cross-loadings were permitted in EFA and retained when they reflected clinically meaningful symptom overlap, consistent with the transdiagnostic nature of the DSM-XC. Model selection was guided by traditional fit indices (Comparative Fit Index [CFI ≥.90], Tucker-Lewis Index [TLI ≥.90], and Root Mean Square Error of Approximation [RMSEA ≤ 0.10]), as well as the clinical interpretability of the factor loadings (e.g., 29, 30). Following the EFA, CFA was conducted on the second subsample (*n* = 1,551) to test the fit of the proposed factor structures identified through EFA.

For Aim 2, to evaluate the fit and clinical applicability of a bifactor model derived from Caspi’s framework of psychopathology, we tested a structure in which all DSM-XC items loaded onto a general psychopathology factor, while three additional specific factors captured unique variance associated with internalizing, externalizing, and thought dysfunction symptoms. Because the model represents a fully specified, *a priori* theoretical structure drawn from the literature and was not informed by the present EFA results, it was evaluated using the full analytic sample (*N* = 3,101) to maximize statistical power. The same estimation methods and model fit criteria used in Aim 1 were applied. Factor loadings were examined to assess the relative strength of the general and specific factors and their alignment with clinically meaningful symptom dimensions.

All exploratory and confirmatory factor analyses were performed using the ‘lavaan’ package (31) in R/RStudio version 4.4.2. Full standardized factor loadings for the final selected EFA solutions are provided in **Supplementary Table 2** to support transparency and reproducibility.

### 2.4 Missing data

DSM-XC surveys were administered as part of routine measurement-based care and were designed to be completed in full or skipped entirely. As partial responses were not recorded, the analytic dataset contained no missing survey data.

## 3 Results

### 3.1 Descriptives

Demographic and clinical characteristics of the full analytic, as well as the EFA and CFA subsamples, are reported in **Supplementary Table 1**. Differences between the EFA and CFA subsamples were evaluated using independent samples t-tests for continuous variables and chi-square tests for categorical variables, and no statistically significant differences were observed (*p* >.05). Descriptive statistics of all DSM-XC items are presented in **Table 1**. Overall, item means indicated a moderate level of symptom burden across the sample. The highest mean scores reflected significant emotional distress, with the most endorsed items being: Item 6: *“Feeling nervous, anxious, frightened, worried, or on edge?”* and Item 2: *“Feeling down, depressed, or hopeless?”* Conversely, the least endorsed items were: Item 13: *“Feeling that someone could hear your thoughts, or that you could hear what another person was thinking?”* and Item 12: *“Hearing things other people couldn’t hear, such as voices even when no one was around?”*. The low mean scores on these items suggest that psychotic experiences were relatively uncommon in this sample.

**Table 1:** Descriptive statistics by item of the DSM-XC (Total sample *N* = 3,101).

		Total sample (*N* = 3,101)	EFA subsample (*n* = 1,550)	CFA subsample (*n* = 1,551)	
Item	Domain name	Mean (*SD*)	Mean (*SD*)	Mean (*SD*)	T-test (*p*-value)
1	Depression	2.08 (1.25)	2.10 (1.26)	2.07 (1.25)	−0.75 (.45)
2	Anger	1.99 (1.29)	2.01 (1.29)	1.97 (1.29)	−0.74 (.46)
3	Mania	0.78 (0.93)	0.78 (0.92)	0.79 (0.94)	0.40 (.69)
4	Anxiety	2.01 (1.18)	2.02 (1.16)	2.01 (1.19)	−0.22 (.82)
5	Somatic Symptoms	1.29 (1.22)	1.31 (1.22)	1.27 (1.23)	−0.74 (.46)
6	Suicidal Ideation	0.41 (0.88)	0.44 (0.89)	0.39 (0.88)	−1.51 (.13)
7	Psychosis	0.13 (0.47)	0.13 (0.46)	0.14 (0.49)	0.39 (.69)
8	Sleep Problems	1.98 (1.47)	1.99 (1.48)	1.96 (1.47)	−0.69 (.49)
9	Memory	1.19 (1.37)	1.20 (1.36)	1.18 (1.39)	−0.32 (.75)
10	Repetitive Thoughts and Behaviors	1.12 (1.16)	1.12 (1.16)	1.12 (1.17)	−0.10 (.92)
11	Dissociation	1.19 (1.38)	1.19 (1.37)	1.19 (1.39)	−0.08 (.94)
12	Personality Functioning	1.38 (1.68)	1.69 (1.36)	1.68 (1.35)	−0.34 (.73)
13	Substance Use	0.45 (0.70)	0.46 (0.71)	0.44 (0.70)	−0.67 (.50)
Item	Symptoms	Mean (*SD*)	Mean (*SD*)	Mean (*SD*)	T-test (*p*-value)
1	Anhedonia	2.06 (1.32)	2.09 (1.33)	2.04 (1.32)	−0.98 (.33)
2	Depression	2.10 (1.34)	2.11 (1.35)	2.09 (1.33)	−0.44 (.66)
3	Irritability	1.99 (1.29)	2.01 (1.29)	1.97 (1.29)	−0.74 (.46)
4	Short-sleep	0.89 (1.20)	0.88 (1.19)	0.89 (1.20)	0.26 (.80)
5	Hyper-activity	0.68 (1.03)	0.67 (1.02)	0.68 (1.04)	0.43 (.67)
6	Anxiety	2.40 (1.30)	2.41 (1.31)	2.40 (1.30)	−0.09 (.93)
7	Panic	1.53 (1.38)	1.53 (1.35)	1.53 (1.40)	−0.01 (.99)
8	Avoidance	2.11 (1.38)	2.12 (1.37)	2.10 (1.39)	−0.48 (.63)
9	Unexplained Pain	1.47 (1.47)	1.50 (1.46)	1.45 (1.49)	−0.89 (.37)
10	Hypochondria	1.11 (1.40)	1.12 (1.40)	1.10 (1.40)	−0.36 (.72)
11	Self-injury	0.41 (0.88)	0.44 (0.89)	0.39 (0.88)	−1.51 (.13)
12	Hallucinations	0.13 (0.55)	0.13 (0.54)	0.13 (0.57)	0.32 (.75)
13	Thought Withdrawal	0.14 (0.56)	0.13 (0.54)	0.14 (0.58)	0.35 (.73)
14	Insomnia	1.98 (1.47)	1.99 (1.48)	1.96 (1.47)	−0.69 (.49)
15	Cognitive Function	1.19 (1.37)	1.20 (1.36)	1.18 (1.39)	−0.32 (.75)
16	Intrusive Thought	1.39 (1.42)	1.42 (1.43)	1.36 (1.41)	−1.03 (.30)
17	Ritualistic Behavior	0.86 (1.27)	0.83 (1.26)	0.88 (1.29)	0.96 (.34)
18	Dissociation	1.19 (1.38)	1.19 (1.37)	1.19 (1.39)	−0.08 (.94)
19	Self-identity	1.63 (1.53)	1.63 (1.53)	1.64 (1.53)	0.23 (.82)
20	Social Function	1.74 (1.44)	1.76 (1.44)	1.71 (1.43)	−0.88 (.38)
21	Alcohol use	0.34 (0.77)	0.34 (0.73)	0.33 (0.80)	−0.29 (.77)
22	Tobacco use	0.55 (1.28)	0.54 (1.27)	0.56 (1.29)	0.53 (.60)
23	Drug use	0.46 (1.10)	0.50 (1.13)	0.43 (1.07)	−1.69 (.09)

Names were shortened to fit table width.

### 3.2 Primary analyses

Conventional factor retention heuristics used to determine the number of factors yielded mixed recommendations. The scree plot favored a 1-factor solution, while the Kaiser-Guttman criterion using the number of eigenvalues over 1.0 suggested a 4-factor solution. The minimum average partial (MAP) criterion suggested an 8-factor solution. Given the lack of convergence across these heuristics, we evaluated solutions ranging from one to eight factors and selected the final model based on a combination of model fit and clinical interpretability. Model fit indices for these solutions are presented in **Table 2**.

**Table 2:** Model fit indices for the exploratory and confirmatory factor analyses.

Exploratory factor analyses						
General solutions	χ^2^	df	p	CFI	TLI	RMSEA [90% CI]
One-factor	3181.00	230	<.01	.92	.91	0.09 [0.088–0.094]
Two-factor	2131.63	208	<.01	.95	.94	0.08 [0.074–0.080]
Three-factor	1191.00	187	<.01	.97	.96	0.06 [0.056–0.062]
Four-factor	863.79	167	<.01	.98	.97	0.05 [0.049–0.055]
Five-factor	563.67	148	<.01	.99	.98	0.04 [0.039–0.046]
Six-factor	399.09	130	<.01	.99	.99	0.04 [0.033–0.041]
Seven-factor	262.15	113	<.01	1.00	.99	0.03 [0.025–0.034]
Bifactor solutions	*χ^2^*	df	*p*	CFI	TLI	RMSEA [90% CI]
Bifactor solutions	*χ^2^*	df	*p*	CFI	TLI	RMSEA [90% CI]
Two-factor	2131.63	208	<.01	.95	.94	0.08 [0.074–0.080]
Three-factor	1191.00	187	<.01	.97	.96	0.06 [0.056–0.062]
Four-factor	863.79	167	<.01	.98	.97	0.05 [0.049–0.055]
Five-factor	563.67	148	<.01	.99	.98	0.04 [0.039–0.046]
Six-factor	399.09	130	<.01	.99	.99	0.04 [0.033–0.041]
Seven-factor	262.15	113	<.01	1.00	.99	0.03 [0.025–0.034]
Eight-factor	171.77	97	<.01	1.00	1.00	0.02 [0.017–0.028]
Confirmatory factor analyses						
Proposed 6-factor solution	1050.58	207	<.01	.98	.97	0.05 [0.048–0.054]
Proposed 5-factor bifactor solution	1671.52	211	<.001	.96	.95	0.07 [0.064–0.070]
Caspi-inspired 4-factor bifactor solution	3178.79	207	<.001	.96	.95	0.07 [0.066–0.070]

df, degree of freedom; CFI, comparative fit index; TLI, Tucker-Lewis index; RMSEA, root mean square error of approximation.

**Figure 1:**
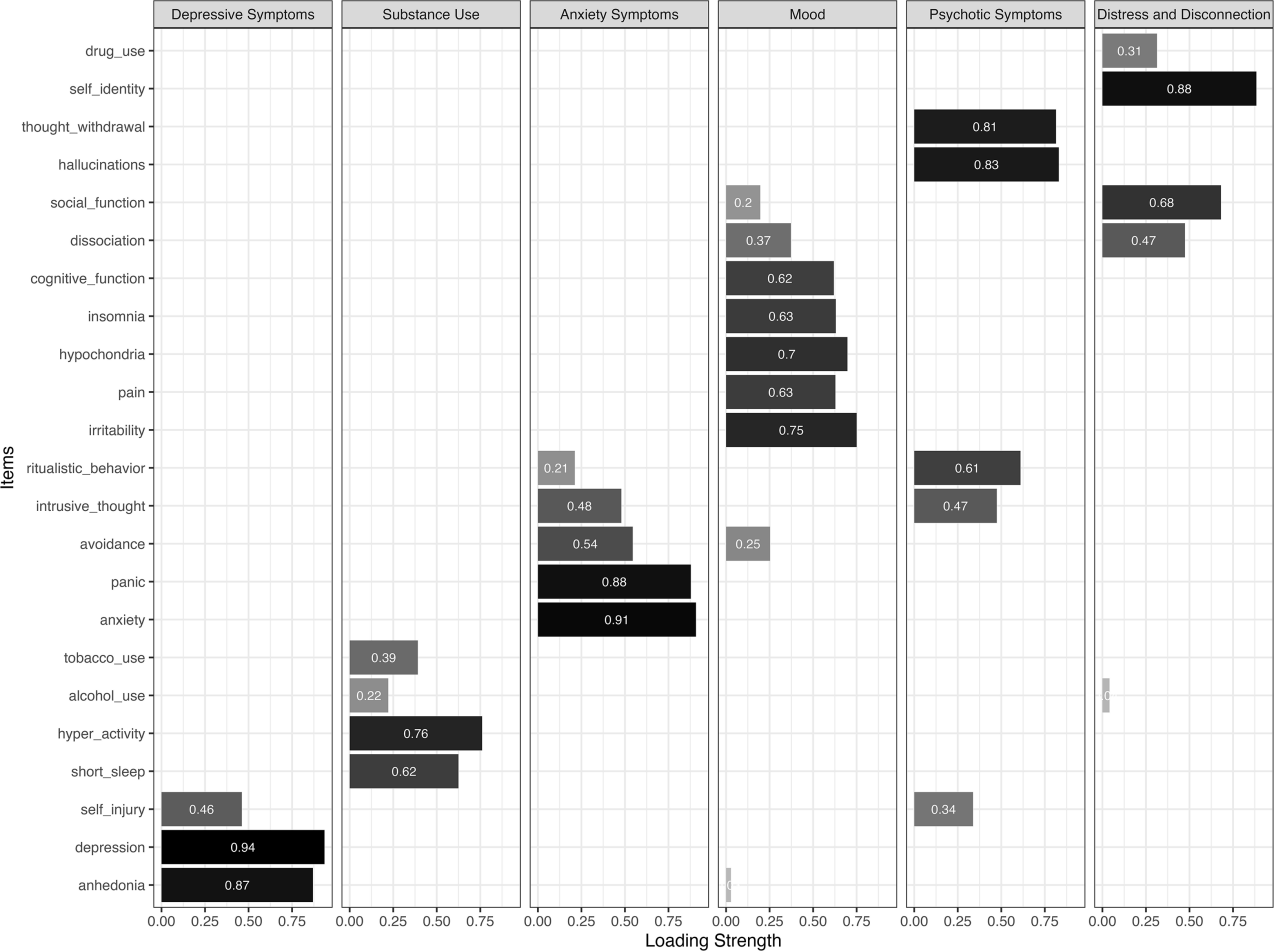
The factor loadings in the 6-factor general solution (CFA estimates reported). Color intensity reflects the magnitude of the factor loadings, with darker shades indicating stronger absolute loadings.

As illustrated in **Figure 1**, the 6-factor solution was determined to provide the best fit, based on relatively liberal criteria for model fit and its interpretability. In this model, all items had salient loadings on at least one factor. Cross-loadings on some items were observed on a second factor and were retained to reflect the transdiagnostic nature of the DSM-XC. The final 6-factor general solution (*χ²*(130) = 399.09, CFI = .99, TLI = .99, RMSEA = 0.04 [0.033–0.041]) identified symptom clusters conceptualized and labeled as ‘Depressive symptoms,’ ‘Substance Use,’ ‘Anxiety Symptoms,’ ‘Mood’, ‘Psychotic Symptoms,’ and ‘Distress and Disconnection.’ This solution effectively captured distinct symptom dimensions, aligning with established constructs in psychopathology (e.g., 32–34).

**Figure 2:**
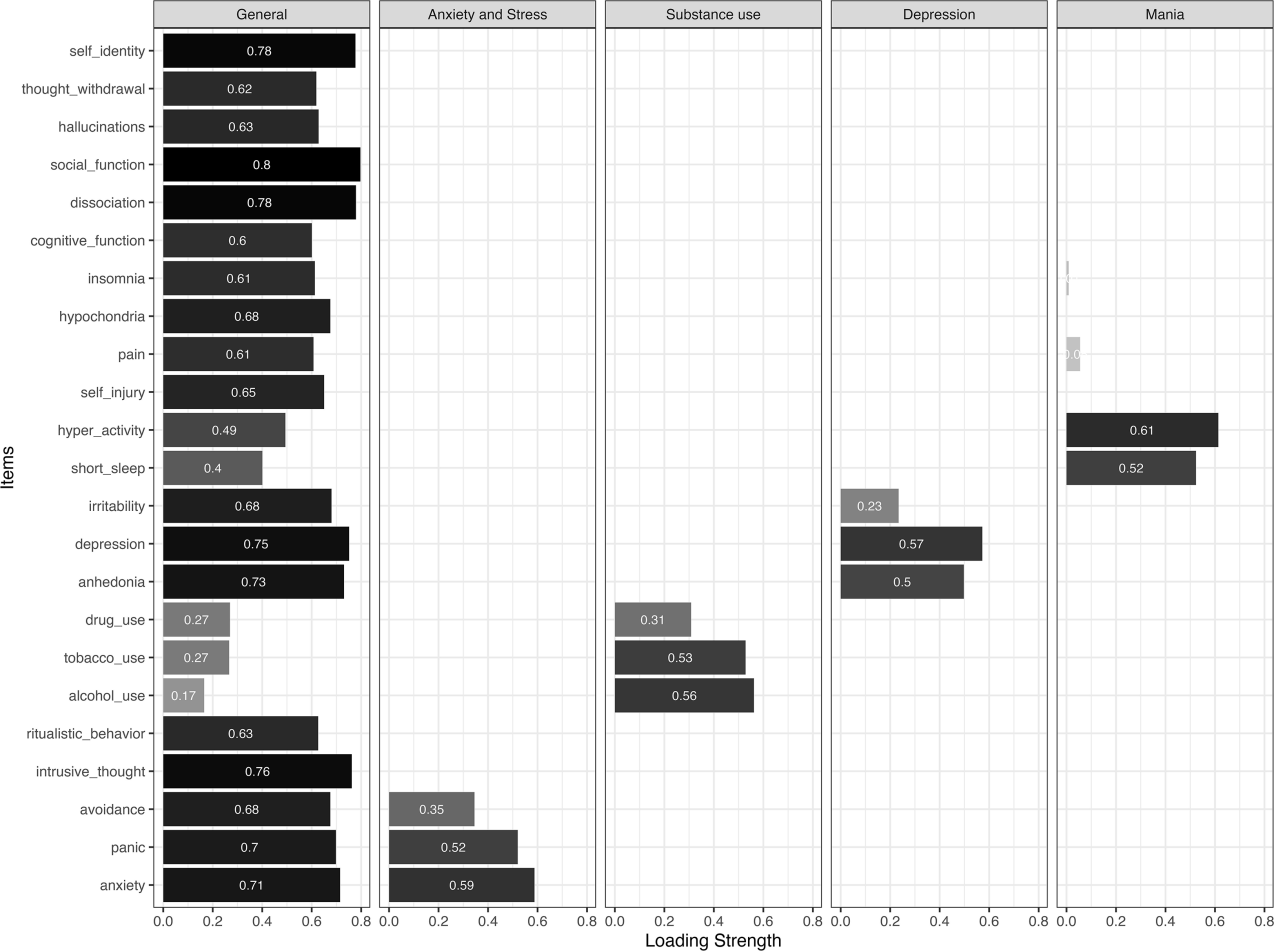
The factor loadings in the 5-factor bifactor solution (CFA estimates reported). Color intensity reflects the magnitude of the factor loadings, with darker shades indicating stronger absolute loadings.

To explore the presence of a general psychopathology factor alongside specific symptom domains, a bifactor EFA was conducted. As shown in **Figure 2**, the best fitting bifactor solution was a 5-factor model (*χ²*(148) = 563.67, CFI = .99, TLI = .98, RMSEA = 0.04 [0.039–0.046]). This model included a general factor representing overall psychopathology burden, along with four specific symptom clusters: ‘Mania’, ‘Anxiety and Stress’, ‘Depression’, and ‘Substance Use’. The general factor was constrained to be orthogonal to the specific factors, and the specific factors were allowed to covary with each other, reflecting shared symptoms across diagnostic categories. This model showed that the general factor accounted for much of the variance in the DSM-XC responses. However, the specific factors provided additional interpretive value, capturing distinct symptom domains that were clinically meaningful.

The CFA subsample was subsequently used to confirm our EFA-derived 6-factor general and 5-factor bifactor solutions in an independent sample. Model fit indices for the CFA solutions are reported in **Table 2**, and the item loadings are illustrated in **Figures 1**, **2** for the general model and bifactor model results, respectively. Although overall model structure was supported, several discrepancies emerged at the item level. Specifically, in the general 6-factor model, the items ‘anhedonia’ and ‘alcohol use’ did not load strongly on the “Mood” and “Distress and Disconnection” factors, respectively, contrary to the findings from the EFA. Additionally, in the 5-factor bifactor model, the items ‘insomnia’, and ‘unexplained pain’ did not load strongly on the “Mania” factor. These discrepancies suggest that certain DSM-XC items may exhibit less stable factor membership across samples, highlighting potential limitations in item specificity that warrant further examination.

**Figure 3:**
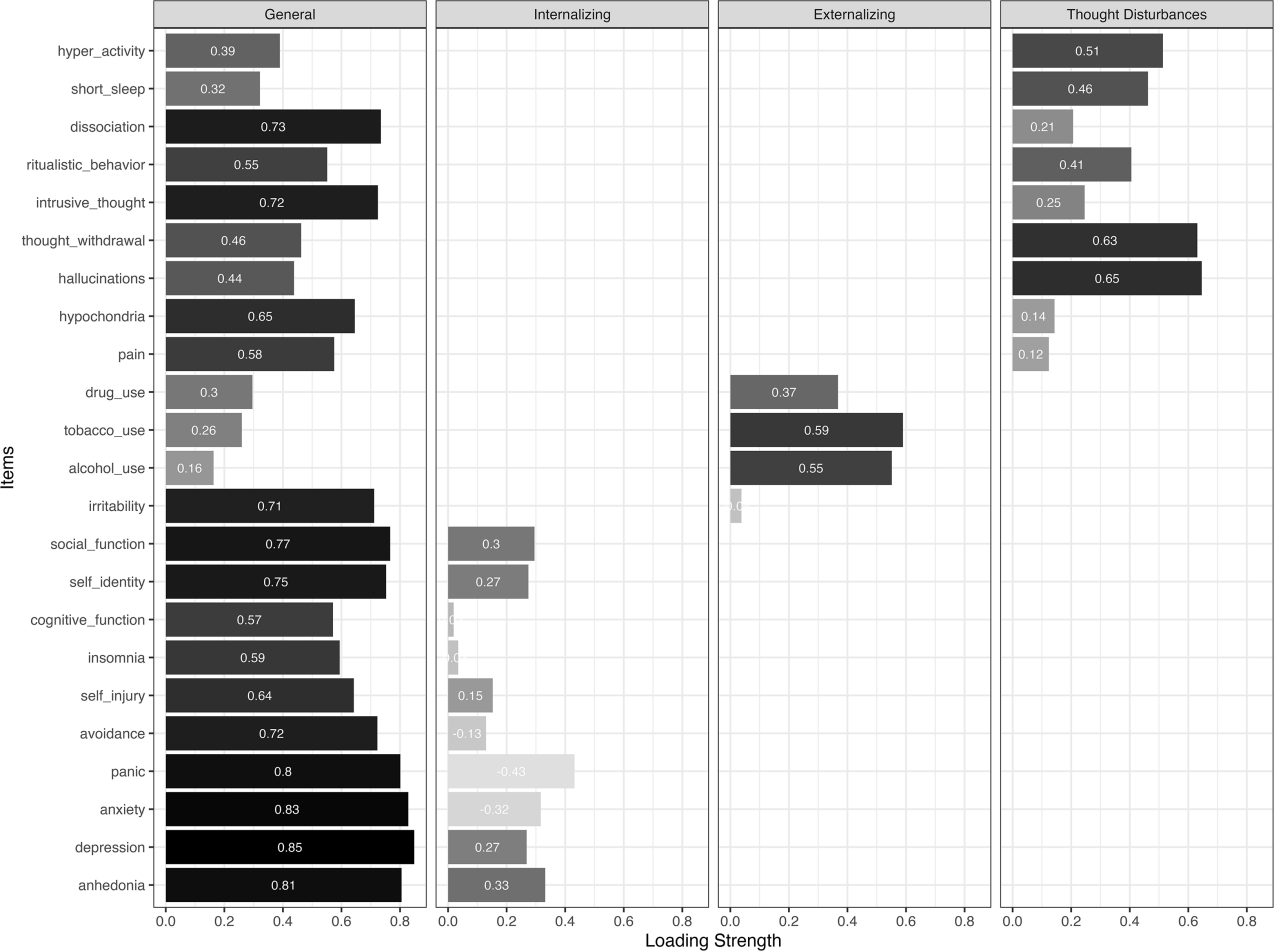
The factor loadings in the Caspi-inspired 4-factor bifactor solution (CFA estimates reported). Color intensity reflects the magnitude of the factor loadings, with darker shades indicating stronger absolute loadings.

Lastly, we tested a theoretically specified bifactor model informed by Caspi’s framework of psychopathology, which includes a general factor along with three specific dimensions: ‘Internalizing’, ‘Externalizing’, and ‘Thought Disturbance’. A trained clinician reviewed the DSM-XC items and assigned them to these domains based on their conceptual alignment. The proposed item assignments were: (1) Internalizing: items assessing depression, anxiety, suicidality, somatic symptoms, and dissociation (DSM-XC items 1, 2, 6, 7, 8, 11, 14, 15, 19, 20); (2) Externalizing: items related to impulsivity, substance use, and anger (items 3, 21, 22, 23); (3) Thought disturbance: items assessing psychosis, cognitive dysfunction, and related symptoms (items 4, 5, 9, 10, 12, 13, 16, 17, 18). This Caspi-inspired bifactor model results demonstrated acceptable fit (*χ²*(207) = 3178.79, CFI = .96, TLI = .95, RMSEA = 0.07 [0.066–0.070]). Item loadings are presented in **Figure 3**. The general factor (“p factor”) showed strong loadings across most DSM-XC items, indicating a substantial shared variance among symptoms. ‘Alcohol use’ had the weakest loading. ‘Externalizing’ and ‘Thought disturbances’ factors showed strong loadings across most items, whereas ‘Internalizing’ demonstrated weak loadings. This pattern suggests that while a general psychopathology factor was robustly supported, the DSM-XC item set does not map cleanly onto the three-domain structure proposed in Caspi’s transdiagnostic framework. These findings and their implications will be explored further in the discussion.

Across analytic approaches, the 6-factor model provided the most coherent and empirically robust representation of the DSM-XC symptom structure in this outpatient psychiatric sample. Although both bifactor models revealed evidence of a general psychopathology dimension, the data more strongly supported a multidimensional symptom architecture.

## 4 Discussion

This study represents one of the most comprehensive investigations of the DSM-XC to date, leveraging the largest known sample of real-world psychiatric outpatients assessed using this measure. By applying an exploratory-confirmatory analytic framework, we were able to rigorously evaluate the latent structure of the DSM-XC and provide new insights into its dimensional validity and clinical utility in routine psychiatric care. Our analyses tested three models, each offering a different perspective on how psychiatric symptoms cluster and co-occur. The EFA identified a 6-factor general solution, with clusters corresponding to ‘Depressive symptoms’, ‘Anxiety’, ‘Mania’, ‘Psychotic Symptoms’, ‘Distress and Disconnection’, and ‘Substance Use’. This structure highlights the multidimensional nature of psychiatric disorders and reflects symptom groupings commonly observed in clinical practice (e.g., 32–34). The prominence of depressive and anxiety symptom clusters, along with the high endorsement of related items, is consistent with epidemiological data showing that mood and anxiety disorders represent the most frequent presentations in outpatient psychiatric care (e.g., 35, 36). This 6-factor model also demonstrates the strongest overall statistical fit to the data, outperforming both bifactor alternatives across conventional fit indices. At the same time, the bifactor models provide important complementary insights into the structure of psychopathology. We explored a 5-factor bifactor solution that included a general psychopathology factor (“p-factor”), alongside specific domains proposed by the EFA (e.g., ‘Anxiety & Stress’, ‘Depression’, ‘Mania’, and ‘Substance Use’). This structure is consistent with transdiagnostic frameworks and underscores the shared variance across symptom domains while preserving their distinctiveness. We also tested Caspi et al’s (5) theory-driven model, which similarly incorporates a general psychopathology dimension along with broader spectra of ‘Internalizing’, ‘Externalizing’, and ‘Thought Disorder’ symptoms. Although neither bifactor model outperformed the 6-factor multidimensional model statistically, both demonstrated acceptable model fit and supported a general psychopathology factor. Taken together, these findings suggest that the DSM-XC can be interpreted from both multidimensional and bifactor perspectives, offering flexibility for clinical screening as well as research aimed at understanding shared and specific mechanisms of psychiatric symptoms.

Among the tested models, the 6-factor solution provides a more granular understanding of psychiatric symptomatology, distinguishing mood disorders (e.g., depressive and anxiety symptoms) from other domains, such as psychotic symptoms and substance use. This structure is consistent with contemporary dimensional models of psychopathology, which conceptualize mental illness as comprising multiple partially independent symptom domains rather than discrete diagnostic categories (e.g., 8, 9). The resulting factors also align with established constructs in psychopathology (32–34), supporting the DSM-XC as a tool capable of capturing the complexity of mental health symptoms across a range of psychiatric diagnoses. Although the 7- and 8- factor general solutions from the EFA demonstrated marginally improved fit indices, these models yielded increasingly fragmented factor structures characterized by small, unstable, or clinically ambiguous dimensions. The 6-factor solution provided a parsimonious representation of the data while achieving good model fit and producing coherent, interpretable symptom domains. Given that the fit indices improve monotonically with increasing model complexity, factor retention was guided by the point at which additional factors no longer contributed substantively meaningful constructs. As such, the six-factor general solution was selected as the optimal balance between statistical accuracy and clinical interpretability. Notably, some discrepancies emerged between the EFA and CFA results. In the CFA, the item ‘anhedonia’ did not load strongly on the “Mood” factor, despite showing a salient loading in the EFA. This could be due to the CFA’s stricter model (37), which might provide a more conservative estimate of its relationship with the Mood factor. Clinically, anhedonia’s weaker loading may reflect its overlap with other mood-related symptoms like irritability, unexplained pain, and insomnia, which dilute its distinctiveness in the CFA model. Similarly, ‘alcohol use’ did not load strongly on the “Distress and Disconnection” factor, which includes drug use, self-identity, social functioning, and dissociation. In this case, alcohol use may function more as a behavioral coping strategy in response to distress rather than as a direct marker of psychological impairment. Additionally, the questions on alcohol use, substance use and smoking are focused more on quantity of use rather than problematic patterns, making them less aligned with the problem-focused nature of the other items in the measure. These findings suggest that substance-related DSM-XC items may capture a distinct behavioral dimension that is clinically meaningful but not fully comparable to internal symptom constructs, warranting potential targeted refinement in future versions of the measure.

The 5-factor bifactor solution offers a complementary perspective on the structure of psychiatric symptoms by modeling both general and specific dimensions of psychopathology. This structure incorporates a general psychopathology factor (5) that accounts for the shared variance across all symptoms while simultaneously acknowledging the distinctiveness of specific symptom clusters. This model provides empirical support for the notion that psychiatric symptomatology reflects both a broad, transdiagnostic liability to distress and more differentiated symptom domains. The general psychopathology factor (“p factor”) represents a higher-order construct that underlies a wide range of psychiatric conditions (5). This factor reflects the commonality of distress and dysfunction across mental health disorders, where individuals with higher levels of general psychopathological burden may be more likely to experience a range of psychiatric symptoms. The bifactor model also allowed for the exploration of specific factors that capture the unique aspects of particular symptom dimensions, while maintaining the independence of the general factor. The structure aligns with transdiagnostic models of psychopathology, which emphasize that mental health conditions are better understood not as discrete categories but rather overlapping dimensions of distress that span across diagnoses (e.g., 6). Allowing the specific symptom clusters to covary in the bifactor model captures the clinical reality that symptoms within the same domain often co-occur and interact. For example, patients with high levels of anxiety may also experience higher levels of distress or substance use (e.g., 38). Of note, in the CFA, ‘unexplained pain’ and ‘insomnia’ items did not load strongly in the “Mania” factor, despite being suggested by the EFA. Clinically, this could reflect the complexity of these symptoms, which, while present in manic episodes, are often seen across a wider range of conditions (e.g., depression), reflecting a broader symptom overlap across psychiatric disorders. These findings illustrate an important obstacle in dimensional modeling: symptoms that are clinically prevalent across disorders may resist clean allocation to a single latent domain, underscoring the need for cautious interpretation and continued refinement of symptom-based measurement tools.

In addition to the 6-factor general and 5-factor bifactor solutions, we also tested a model inspired by Caspi and colleagues (5). Although this model demonstrated acceptable fit, it did not perform as strongly as the 6-factor general model. Inspection of the factor loadings further indicated that the internalizing factor was weakly represented, with several items showing low loadings, suggesting limited empirical support for this domain as operationalized within the DSM-XC. Similarly, the externalizing factor was driven primarily by substance use items, with relatively few indicators contributing strongly highlighting a structural limitation of the measure for capturing this domain. Future iterations of the measure could benefit from incorporating additional content relevant to externalizing symptoms. For example, while the child and parent-informant versions of the DSM-XC include an item assessing ADHD symptoms, the adult version does not, potentially reflecting a historical under-recognition of ADHD at the time of the measure’s development. In addition to measurement limitations of the DSM-XC, the high degree of comorbidity in the present psychiatric sample may further blur distinctions between internalizing and externalizing symptom domains. In highly comorbid clinical populations, symptom dimensions may be more tightly coupled, reducing the empirical separability of hierarchical spectra that are more readily observed in general population samples. Furthermore, Caspi’s model was derived from a general population sample, not a psychiatric cohort, which may help explain why it did not fit as well in the current study.

Several limitations should be noted. First, the sample was predominantly Caucasian and female, which may limit the generalizability to more diverse populations. Future research should replicate these findings in more heterogeneous samples. Second, the study setting was limited to private practice psychiatric outpatients, where some symptom domains (e.g., psychosis) are less prevalent than in inpatient, community health, or emergency settings. Third, the cross-sectional design precluded examination of how symptom clusters and factor structure may evolve over time. Longitudinal studies are needed to assess temporal stability and predictive validity. Additionally, the DSM-XC was administered as part of routine clinical care rather than at a standardized time point. Consequently, the timing of questionnaire completion relative to treatment initiation varied across patients, and symptom severity may therefore reflect different stages of treatment. Future studies using prospective designs with standardized assessment intervals would help clarify how timing influences symptom profiles and factor structure. Finally, although bifactor models have strong theoretical relevance to transdiagnostic frameworks, their clinical utility may be limited by the complexity of interpreting both a general psychopathology factor along with specific symptom domains. It may be challenging to apply these models in routine clinical practice, where a simpler, more straightforward symptom classification system might be preferred. However, as research continues to refine the understanding of psychopathology, bifactor models may provide a valuable framework for understanding the shared and unique contributions of psychiatric symptoms to the overall burden of illness. Future work should explore the clinical utility of the identified factor structures, including how they can be integrated into routine practice to support screening, symptom monitoring, and treatment planning. Despite these limitations, understanding the latent factor structure of the DSM-XC has important clinical implications, as the measure becomes more widely used in psychiatric settings. Identifying symptom clusters can guide clinicians in selecting follow-up assessments or clinical interviews and help determine whether a broad, transdiagnostic model (e.g., 5-factor bifactor model) or a more domain-specific approach (e.g., 6-factor model) is most useful. In clinical practice, where time and resources are limited, using empirically supported symptom clusters can improve screening efficiency, track symptom changes, and guide personalized care, ultimately enhancing the DSM-XC’s utility in diagnosis and monitoring in the real-world.

### 4.1 Conclusion

Our findings support the DSM-XC’s utility as a dimensional tool for assessing psychiatric symptoms in real-world settings. To our knowledge, this is the largest study to date examining the DSM-XC in a psychiatric outpatient population, offering robust insights into its latent structure at scale in the real-world. Symptom clustering was consistent across models, reflecting both general and specific psychopathology domains and reinforcing the relevance of transdiagnostic, dimensional approaches to psychiatric assessment. The 6-factor general and 5-factor bifactor models offered the strongest fit. The former supported a multidimensional organization of symptoms, whereas the latter indicated the presence of a general psychopathology factor. The Caspi-inspired model yielded a more simplified but less optimal structure. Together, these results highlight the DSM-XC’s value for clinical screening and psychiatric research, supporting its use in guiding follow-up assessment and individualized care. Future work should examine longitudinal stability, sensitivity to change, and how dimensional profiles derived from the DSM-XC integrate into routine clinical decision-making.

## Data Availability

The data analyzed in this study is subject to the following licenses/restrictions: The data used in this study were derived from real-world electronic health records (EHRs) containing sensitive patient information collected in clinical settings. Due to the sensitive nature of these data and organizational policies designed to protect patient privacy, the dataset cannot be shared publicly. Requests to access these datasets should be directed to research@osmind.org.
