# Supplementary material for: Psychometric evaluation of the DSM-5-TR-Level 1 Cross-Cutting Symptom Measure: transdiagnostic factor analysis in a real-world psychiatric outpatient sample
Source: Front Psychiatry. 2026 Apr 10;17:1680352. doi: 10.3389/fpsyt.2026.1680352 (PMC13106460; doi:10.3389/fpsyt.2026.1680352)
Supplement: Supplementary file 1 [file Table1.docx]

**Supplementary Tables**

**Table 1.** Demographic descriptives by subsamples

|  | **Total Sample  (*N* = 3,101)** | **EFA Subsample (*n* = 1,550)** | **CFA Subsample  (*n* = 1,551)** | **T-test / chi-square; *p*-value** |
| --- | --- | --- | --- | --- |
| Symptoms |  |  |  |  |
| Age (Mean, *SD*) | 38.8 (13.8) | 38.6 (13.7) | 39.0 (14.0) | *t* = 0.82; *p* = .41 |
| Sex (n, %) |  |  |  | *ᶍ^2^* = 0.006; *p* = .94 |
| Females | 1959 (63.2%) | 982 (63.4%) | 977 (63.0%) |  |
| Males | 966 (31.1%) | 482 (31.1%) | 484 (31.2%) |  |
| NA | 176 (5.7%) | 86 (5.8%) | 90 (5.5%) |  |
| Race (n, %) |  |  |  | *ᶍ^2^* = 2.33; *p* = .80 |
| White | 2020 (65.1%) | 1027 (66.3%) | 993 (64.0%) |  |
| American Indian or Alaska | 18 (0.58%) | 9 (0.58%) | 9 (0.58%) |  |
| Asian | 118 (3.81%) | 56 (3.61%) | 62 (4.0%) |  |
| Black or African American | 109 (3.51%) | 48 (3.10%) | 61 (3.93%) |  |
| Native Hawaiian or   Other Pacific Islander | 26 (0.84%) | 13 (0.84%) | 13 (0.84%) |  |
| Mixed Race | 110 (3.55%) | 55 (3.55%) | 55 (3.55%) |  |
| NA | 700 (22.6%) | 358 (22.1%) | 342 (23.1%) |  |
| Ethnicity (n, %) |  |  |  | *ᶍ^2^* = 0.92; *p* = .34 |
| Hispanic or Latino | 255 (8.22%) | 120 (7.74%) | 135 (8.70%) |  |
| Not Hispanic or Latino | 2262 (72.9%) | 1141 (73.6%) | 1121 (72.3%) |  |
| NA | 584 (18.8%) | 289 (18.6%) | 295 (19.0%) |  |
| Number of diagnoses (Mean, *SD*) | 6.04 (4.4) | 5.97 (4.15) | 6.10 (4.63) | *t* = 0.77; *p* = .44 |

**Table 2.** Exploratory Factor Analysis loadings for the Final 6-factor General and 5-factor Bifactor Solutions
*Note:* Loadings > .30 are shown in bold and marked with an asterisk (*) indicating salient loadings retained for interpretation

|  | **Number of factors** | | | | | | | | | | |
| --- | --- | --- | --- | --- | --- | --- | --- | --- | --- | --- | --- |
|  | **General** | | | | | | **Bifactor** | | | | |
| **Symptoms** | 1 | 2 | 3 | 4 | 5 | 6 | 1 | 2 | 3 | 4 | 5 |
| anhedonia | **0.47*** | -0.07 | -0.002 | **0.51*** | -0.002 | 0.17* | 0.75* | 0.03 | -0.01 | **0.46*** | -0.001 |
| depression | **0.74*** | -0.003 | 0.03 | 0.33 | 0.13 | -0.01 | 0.78* | 0.14* | -0.005 | **0.45*** | -0.002 |
| irritability | 0.25* | 0.16* | 0.14* | **0.46*** | -0.06 | 0.01 | 0.62* | 0.13* | 0.01 | **0.29*** | 0.25* |
| short-sleep | 0.003 | **0.55*** | -0.006 | 0.27 | 0.003 | -0.18* | 0.29* | 0.01 | 0.12 | 0.02 | **0.57*** |
| hyper-activity | -0.10 | **0.60*** | 0.01 | 0.20 | 0.13 | 0.01 | 0.39* | 0.01 | 0.26* | -0.11 | **0.49*** |
| anxiety | 0.11 | 0.03 | **0.85*** | 0.04 | 0.005 | -0.04 | 0.64* | **0.66*** | 0.01 | 0.14* | 0.02 |
| panic | 0.001 | 0.03 | **0.82*** | -0.01 | 0.12* | -0.01 | 0.63* | **0.62*** | 0.01 | -0.02 | 0.003 |
| avoidance | 0.003 | -0.05 | **0.44*** | **0.32*** | -0.001 | 0.16* | 0.63* | **0.30*** | -0.02 | 0.10 | 0.03 |
| unexplained pain | -0.18 | 0.002 | 0.003 | **0.78*** | 0.001 | -0.02 | 0.55* | -0.04 | -0.29* | -0.004 | **0.41*** |
| hypochondria | 0.06 | -0.003 | 0.01 | **0.58*** | 0.18* | -0.03 | 0.64* | 0.01 | -0.23* | 0.01 | 0.28* |
| self-injury | **0.47*** | 0.19* | -0.02 | 0.01 | **0.47*** | 0.02 | 0.66* | 0.10 | 0.14* | 0.02 | -0.01 |
| hallucinations | 0.003 | 0.21 | -0.11 | 0.01 | **0.74*** | -0.04 | 0.53* | -0.01 | 0.002 | -0.51* | 0.12 |
| thought withdrawal | 0.01 | 0.19 | 0.04 | -0.12 | **0.80*** | -0.01 | 0.58* | 0.10 | 0.05 | -0.55* | 0.03 |
| insomnia | 0.11 | 0.10 | 0.02 | **0.53*** | 0.02 | -0.05 | 0.53* | 0.01 | -0.13 | 0.14* | **0.32*** |
| cognitive function | -0.05 | 0.03 | -0.04 | **0.50*** | 0.20* | 0.05 | 0.56* | -0.05 | -0.15* | -0.08 | 0.24* |
| intrusive thought | 0.19* | 0.01 | **0.33*** | -0.01 | **0.56*** | 0.06 | 0.76* | 0.29* | 0.01 | -0.18 | -0.09 |
| ritualistic behavior | -0.09 | -0.002 | **0.33*** | 0.01 | **0.51*** | 0.08 | 0.61* | 0.25* | -0.04 | -0.35* | -0.04 |
| dissociation | -0.004 | -0.03 | 0.15* | **0.36*** | 0.29* | **0.31*** | 0.78* | 0.09 | 0.03 | -0.08 | -0.02 |
| self-identity | 0.11 | 0.01 | 0.01 | 0.44 | 0.12 | **0.53*** | 0.82* | -0.02 | 0.22* | 0.13 | -0.01 |
| social function | 0.18 | 0.03 | -0.02 | **0.56*** | -0.004 | **0.47*** | 0.80* | -0.03 | 0.19* | 0.25 | -0.02 |
| alcohol use | 0.03 | **0.44*** | 0.01 | -0.12 | -0.10 | **0.36*** | 0.16* | 0.003 | **0.56*** | 0.13 | 0.01 |
| tobacco use | -0.02 | **0.36*** | -0.05 | 0.01 | 0.10 | 0.27* | 0.28* | -0.04 | **0.38*** | -0.03 | 0.09 |
| drug use | -0.03 | 0.28* | -0.01 | -0.01 | 0.17 | **0.33*** | 0.35* | -0.01 | **0.37*** | -0.07 | -0.01 |

**Table 3.** Internal consistency (Cronbach’s Alpha) for DSM-XC Domain Scores

*Note:* Internal consistency is not applicable for domains represented by a single item

| **Domain Name** | **Cronbach’s ɑ** |
| --- | --- |
| Total | .90 |
| Depression | .87 |
| Anger | – |
| Mania | .56 |
| Anxiety | .83 |
| Somatic Symptoms | .62 |
| Suicidal Ideation | – |
| Psychosis | .60 |
| Sleep Problems | – |
| Memory | – |
| Repetitive Thoughts and Behaviors | .65 |
| Dissociation | – |
| Personality Functioning | .81 |
| Substance Use | .36 |
